# How equitable is digital rehabilitation for people after stroke? A systematic review using an equity approach

**DOI:** 10.3389/fdgth.2025.1544754

**Published:** 2025-06-24

**Authors:** Rachel C. Stockley, Yasemin Hirst, Chantelle Hayes, Kimberley E. Watkins, Peter C. Goodwin

**Affiliations:** ^1^ Stroke Research Team, School of Nursing and Midwifery, University of Central Lancashire, Preston, United Kingdom; ^2^ Physiotherapy Department, Derbyshire Community Health Services NHS Foundation Trust, Derby, United Kingdom; ^3^ College of Saint Scholastica, Duluth, MN, United States; ^4^ Department of Health Professions, Manchester Metropolitan University, Manchester, United Kingdom

**Keywords:** stroke, rehabilitation, digital, digital health, equity, PROGRESS-Plus

## Abstract

**Introduction:**

Stroke is the largest global cause of adult neuro-disability. Health inequities increase the risk of stroke and are likely to influence overall recovery. Rehabilitation after stroke seeks to restore function and independence and may utilise digital technologies to augment usual care. This study systematically investigates the reporting of equity factors in digital stroke rehabilitation research.

**Methods:**

This systematic review examined equity factors contained in the PROGRESS-Plus framework in a random sample of clinical trials of technologies used as part of stroke rehabilitation published in 2011–2021. Four reviewers double-screened titles and abstracts of 14,724 papers. A random selection was carried out across all potentially eligible papers (*n* = 821) and 135 papers were reviewed for data extraction. Each study was coded with 36-point PROGRESS-plus criteria for inclusion, exclusion, and baseline characteristics. ANOVA and multivariable linear regression were used to assess the variation in PROGRESS-Plus reporting by year of publication, location, type of technology used, intervention target, number of comparison groups and sample size.

**Results:**

87 studies were included with a mean PROGRESS-Plus score of 7.05 (*SD* *=* 2.06), minimum score of 0 and maximum score of 14. Despite their importance to health outcomes, education, social capital and socioeconomic status were reported by less than 5% of studies. The most commonly reported equity factors were age, disability and gender. There were no significant differences in reporting by technology used, target of the intervention (upper or lower limb), sample size, location, number of comparison groups and sample size. Variation in equity reporting was not explained through multiple linear regression factors. There was a small positive correlation between the year of publication and the PROGRESS-Plus score (*r* = .26, *n* = 87, *p* < 0.05).

**Discussion:**

Few studies of digital rehabilitation interventions considered several key equity factors, including those recognised to precipitate digital exclusion and influence health outcomes. An encouraging finding was that more recent work was slightly more likely to report equity factors, but future research should ensure complete reporting of equity factors to ensure their findings are applicable to clinical populations.

**Systematic Review Registration:**

https://www.crd.york.ac.uk/PROSPERO/view/CRD42024504300, PROSPERO/identifier, CRD42024504300.

## Introduction

Stroke is the second leading cause of death and the third leading cause of disability, worldwide, affecting over 12 million people each year (1). Globally one in four people will have a stroke in their lifetime (2) and disability after stroke accounts for 143 million disability-adjusted life-years (DALYs) (1).

Rehabilitation is a key priority for stroke services (1). Rehabilitation provides a set of interventions that support people to be as independent as possible in daily life and enable their participation in life roles such as education, work, recreation and caring for others (3). Accordingly, rehabilitation is a key priority for global stroke services (1, 3) and significant focus has been placed upon research to evaluate rehabilitative interventions to maximise recovery after stroke. In the last 20 years, many studies have sought to evaluate the potential of a range of digital technologies to provide interventions as part of rehabilitation. These digital health technologies (DHT) comprise a broad range of products including software applications (apps), wearable sensors, telehealth, robotics, non-invasive stimulation, brain-computer interfaces and virtual reality systems which may be standalone or combined with other interventions (4). Digital health technologies complement, but are qualitatively different to conventional rehabilitation, as they require hardware, connectively and digital skills to use successfully. However, DHT are becoming more widely used throughout healthcare and rehabilitation as they present an attractive solution to overcome challenges accessing rehabilitation due to limitations in geography (e.g., telehealth), and staff availability (e.g., apps). They also offer novel approaches that cannot be replicated by traditional approaches (e.g., brain-computer interfaces and electrical stimulation), provide real time feedback to clinicians and patients, as well as offering engaging and motivational ways to undertake the significant doses of training required to optimally recover (e.g., virtual reality). However, it is recognised that some people in the general population are excluded from using digital resources as they lack access, do not have the skills or cannot afford to (5). The factors contributing to this digital exclusion are multi-faceted and emergent, but lower socioeconomic status, disability, older age and less education are commonly associated with reduced use of digital media (6). In people receiving rehabilitation, pre-existing levels of digital exclusion can also be heightened by clinicians who may use assumptions about who will be able to use DHT, effectively acting as “gatekeepers” by only offering interventions to selected groups or individuals (7–10).

In addition to digital exclusion, research into rehabilitative DHT after stroke is also likely to be compounded by established inequities in who participates in clinical research (11). Trials have often recruited narrow, homogeneous populations to limit the impact of uncontrolled factors upon outcomes, to reduce variance within the sample to minimise “noise” and to make sample sizes manageable and therefore equitable to research funders (11, 12). Practical difficulty accessing research sites for people with poor mobility, availability of research materials in other languages and formats, and the absence of support for people who may lack the capacity to consent can also affect the opportunity to participate (13). These factors are particularly pertinent for research in people who have had a stroke; conservative estimates indicate that around 70% of people after a stroke will have reduced mobility, 20% will have difficulties with communication and 40% may have significant cognitive deficits (14).

The cumulative effect of both digital and research exclusion has significant consequences for both the external validity of the findings of the research and the recovery of people after stroke. If populations recruited into trials bear little resemblance to the clinical target population, it produces a disconnect between the findings of DHT stroke research based on trial populations and clinical reality. Restricting access to participation in research deprives individuals and specific groups of opportunities to access novel interventions which may confer additional benefits to the usual care they receive and could be potentially interpreted as discriminating against some groups (11, 15, 16). This produces a significant barrier to the confident application of research findings to clinical practice as marked differences between clinical and research populations mean that benefits and harms observed in trials may not translate into those seen in clinical practice and different responses to interventions from different subgroups may be missed. This ultimately wastes investment in research and means that clinical populations may not receive the right intervention at the right time to optimise their recovery. Furthermore, incomplete understanding of who may benefit from specific interventions may skew the planning and commissioning of stroke services, perpetuating systemic inequities at a system level.

The positive effects of ensuring inclusion in research have become more accepted in recent years, with both journals and research funders recognising the importance of broad consideration of inclusion and accurate reporting in research (17, 18). The importance of reporting equity factors in research is explicitly recognised in the freely available Cochrane Collaboration's PROGRESS-Plus framework (19). This pragmatic framework highlights key social determinants of health and factors that are recognised to influence health opportunities and inclusion, including: place of residence, race/ethnicity, occupation, gender, religion, education, social capital, socioeconomic status and other factors such as personal characteristics (e.g., disability), features of relationships and time-dependent relationships (19). It is the predominant tool used to capture dimensions of health equity and its use has been growing in recent years (20), although it is largely used in public health settings, rather than applied clinical research.

We believe that whilst the research evaluating the ever-expanding use of DHT in stroke rehabilitation is rapidly increasing, it has the potential to heighten inequities because it combines three areas where inequities are present, namely: inequities from digital exclusion, inequities related to a range of impairments produced after stroke and established health inequalities that increase the risk of having a stroke. This systematic review sought to utilise the PROGRESS-Plus framework to understand which equity factors are reported in DHT rehabilitation trials and considered when including or excluding potential participants into DHT rehabilitation trials, and to examine any relationship between the type of DHT being evaluated and use and reporting of equity factors.

## Methods

A systematic review approach with a random paper selection was adapted from Wilson and colleagues (21). The project was registered with PROSPERO (ref: CRD42024504300).

### Research question

Do DHT in randomised controlled trials in stroke rehabilitation report participant characteristics?

a. What are the equity factors that are most frequently used to include/exclude participants in physical rehabilitation stroke trials?
b. What are the indicators of reporting a greater number of equity factors in physical rehabilitation stroke trials?

### Search strategy

A search string was derived using PICO (Patient/population, intervention, comparator and outcome). Medline (Ovid), Embase (Ovid), CINAHL (EBSCOhost) and Cochrane Library. The search strategy was developed by an information specialist with input from the review team and included search terms and subject headings relating to physical therapy, rehabilitation, stroke and clinical trials (Table 1). The search strategy was adapted for use in each database. Search terms for stroke were taken from the Cochrane Stroke Strategy Search filters and were used in Medline, Embase and CINAHL to identify relevant study designs (22). We used the Cochrane Highly Sensitive Search Strategy for identifying randomized trials (23). The full search strategy used for each database can be found in Supplementary Materials. Searches were limited by date from 2011 to ensure that the technologies being evaluated were still likely to be current. In addition, 2011 coincided with or just preceded the establishment of two key forms of DHT, telerehabilitation and virtual reality, for rehabilitation after stroke [evidenced by their inclusion in Cochrane reviews (24, 25)] but provided a sufficiently large sampling window (exceeding a decade) to ensure relevance to current practice. Papers not written in English were not included due to the absence of funding to support translations. The results from each database were imported into EndNote and duplicates were removed by the information specialist using EndNote functionalities and manually. The deduplicated records were then imported into the Rayyan AI web application for screening (26).

**Table 1:** PICO used for developing search strategy.

Characteristics	Inclusion criteria
Patient/Population	adults after stroke
Intervention	receiving any form of physical rehabilitation
Comparison	comparator usual care or another intervention
Outcome	no outcomes

### Study eligibility

Studies were eligible if they used (1) a digital health technology defined by NICE guidance (4), but we also sought terms that were not explicitly listed by NICE such as applications (apps) and m-health. Consequently, screening included technologies that were applications, utilised robotics, virtual reality, brain computer interfaces, wearable sensors, robotics, exoskeletons, digital treadmills, peripheral or neuromuscular electrical stimulation or provided brain stimulation (magnetic or electrical), (2) employed randomised controlled trial (RCT; pilot or full trial) methodology, (3) included adult (≥18 years) patients/participants, (4) participants had a confirmed diagnosis of stroke, (5) evaluated a rehabilitation intervention, (6) were published in English, and (7) included participant characteristics including inclusion and/or exclusion criteria. The studies were excluded if they were (1) protocols, (2) controlled trials with no clear randomisation, (3) crossover trials where participants act as their own controls or (4) no participant characteristics were reported.

### Screening of the search results

Four reviewers carried out abstract and title screening in a three-step process. Firstly, a minimum of two reviewers blind-screened titles and abstracts of stroke rehabilitation RCTs to exclude ineligible research studies using Rayyan AI (26). All eligible articles were coded with the type of technology used developed from NICE definitions (4). Secondly, where the type of technology was not clear or not explicitly listed within the NICE definition, reviewers achieved consensus on the classification through discussion. At the end of each blind review process, the reviewers assessed conflicts together. Thirdly, disagreements were resolved through discussion with the wider team. Due to the number of the papers identified at screening stage and for pragmatic reasons, the authors only coded the reasons for exclusions which were deliberate and joint decisions made by two reviewers. We did not assess inter-rater agreement.

### Sample size calculations

*A priori* sample size calculations indicated a minimum of 84 papers were required to have 80% power at a 5% significance level to test for differences in the number of items included on the PROGRESS-Plus by year of publication, area of the body which was the primary target of intervention (e.g., upper or lower extremity or both), sample size, and DHT categories. An additional 20% (*n* = 16) to account for attrition (e.g., manuscript cannot be accessed, abstract or poster presentation, not in English) was also included (27) determined that 100 full papers should be randomly selected and extracted from the overall data.

### Data extraction

Papers were selected for data extraction using a random number generator (27).

All papers identified at the end of the screening process were extracted onto a custom Excel sheet and subjected to random sampling. If randomly selected and eligible for data extraction, they were included in the final dataset (see Supplementary Data). Data for the intervention category, the type of digital intervention used, year of publication, sample size, primary and secondary outcomes, the number of trial arms were extracted from each paper. If a paper was randomly selected from the unclear DHT category, the type of DHT was identified and re-coded to be included in its respective category. PROGRESS-Plus criteria were extracted from both inclusion and exclusion criteria and baseline characteristics. The criteria include 12 categories (place of residence, Race/ethnicity/culture/language, occupation, gender/sex, religion, education, socioeconomic status, social capital, Plus Age, Plus Disability, features of relationship and time-dependent relationships). With the 3 sub-categories (inclusion criteria, exclusion criteria and baseline characteristics) equated to a total number of 36 variables for PROGRESS-Plus (19). Each item was coded 1 if any of the characteristics were reported and 0 if they were not reported. The coding structure is included in the Supplementary Materials including PROGRESS-Plus definitions. Our primary outcome was the total PROGRESS-Plus score.

### Assessment of bias

The focus of the current review is to understand which groups of patients participate in research, rather than to consider the quality or findings of the research studies. Therefore, no formal assessment of bias for the included studies was undertaken, no judgements were made on effectiveness of the interventions included in the studies nor any meta-analyses undertaken.

### Data analysis

A total equity score for each paper based on the PROGRESS-Plus Criteria was computed. Descriptive tables and figures were generated to illustrate basic details for papers (location, year of publication, size, design and target) and the participant characteristics reported in each study.

The one-way analysis of variance (ANOVA) was used to determine whether there are any statistically significant differences in the computed equity score between the year of publication (2011–2015, 2016–2019, 2020–2021), extremity (upper vs. lower limb), the number of groups compared in a paper (2/3/4 groups), sample size (30 or less = 0; 31 or more participants = 1). For statistical analysis, the digital technologies were grouped to reduce the number of categories to be able to make meaningful comparisons, namely (1) technologies that provide physical support to undertake training, i.e., balance platforms, exoskeleton, robotics and treadmill, (2) forms of stimulation, i.e., magnetic stimulation, brain stimulation and theta stimulation, (3) forms of electrical stimulation, i.e., neuromuscular stimulation, electrical stimulation and peripheral stimulation (4) sensors and feedback (i.e., biofeedback, brain-computer interface, wearables), (5) technologies that provide remote activity typically as part of self-management/off-site, i.e., telehealth and apps, and (6) forms of engaging training using Virtual Reality (VR). The associations between categorical variables were assessed using ANOVA and continuous variables were assessed using Spearman's rank-order correlations. A multiple linear regression test was used to assess factors associated with the use of PROGRESS-Plus scores. However, the model was not significant and not reported in this paper. All statistical analyses were carried out using IBM SPSS 29 with a *p*-value less than 0.05 to indicate statistical significance.

## Results

### Study selection

**Figure 1:**
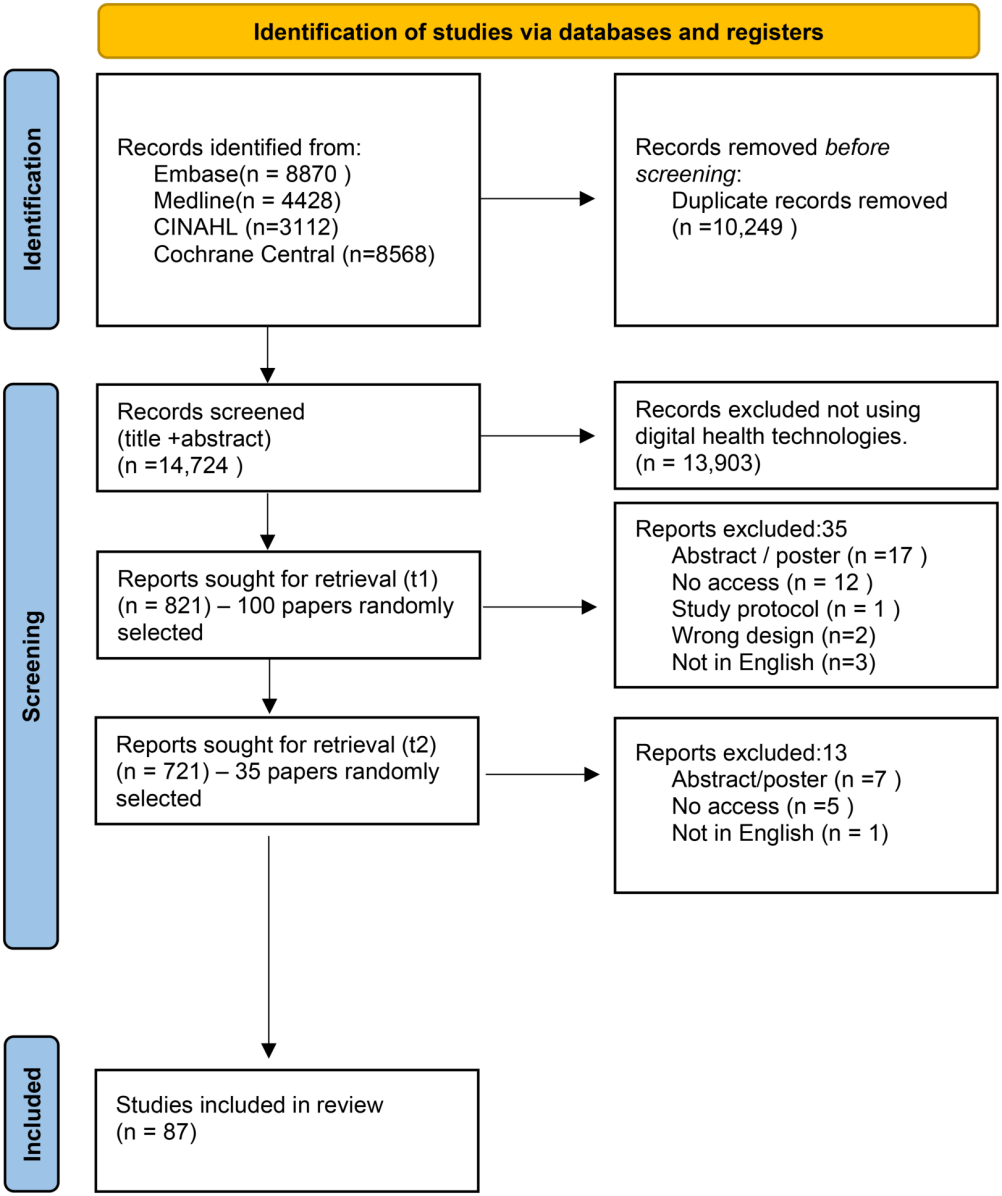
PRISMA diagram to show article flow through the study.

A total number of 14,724 papers were identified using the search strategy published between December 2011 and December 2021. Of those, 13,903 papers (94.4%) were excluded at title and abstract screening if they did not meet the eligibility criteria. This left a total of 821 papers which were eligible for full paper review ( Figure 1 ).

The random selection of papers was carried out twice to meet the minimum sample size. Out of the first 100 papers selected (100/821) at Time 1 (t1), 35 papers were ineligible for full paper review. During the second random selection phase (Time 2, t2), 35 papers were included from 721 papers (excluding the selection from the first random sample) and 13 papers were excluded. The reasons for exclusion at t1 and t2 were recorded and reported on the PRISMA flow diagram ( Figure 1 ). These were not having access to the publication, not in English, poster/abstract publications, wrong study design (e.g., study protocol), or not a digital intervention.

A random selection was carried out across all potentially eligible papers (*n* = 135) and 48 papers were excluded at the full paper review stage. In total, 87 out of 821 papers (10.6%) were included for data extraction (see Figure 1), exceeding the minimal sample required (*n* = 84).

### Characteristics of the DHT stroke rehabilitation interventions included

4271 participants were included across the 87 randomly selected papers with a mean average of 49 participants in a study (minimum 5 participants and maximum 770 participants; SD: 86.25) (see Table 2). Most papers were published in Asia (67.8%), followed by Europe (16.1%) and North America (9.2%). Only three papers each were published respectively in South America (3.4%) and Africa (3.4%) among the randomly selected papers. About half of the interventions targeted the lower extremity (49.4%) for stroke rehabilitation whereas 46% targeted the upper extremity and 4.6% targeted both extremities.

**Table 2:** Study characteristics of the random selection of papers (*n* = 87).

Characteristics	*N* (%)
Total	87 (100)
Location	
Europe	14 (16.1)
South America	3 (3.4)
North America	8 (9.2)
Asia	59 (67.8)
Africa	3 (3.4)
Year of publication	
2011–2015	31 (35.6)
2016–2019	39 (44.8)
2020–2021	17 (19.5)
Intervention target	
Lower extremity	43 (49.4)
Upper extremity	40 (46.0)
Both	4 (4.6)
Number of comparison groups (including control)	
Two groups	74 (85.1)
Three groups	11 (12.6)
Four groups	2 (2.3)
Sample size (categorical)	
30 or less participants	45 (51.7)
31 or more participants	42 (48.3)
Mean (standard deviation)	
Sample size [range 5–770]	49.09 (86.25)
Total PROGRESS- plus score [range 0–36]	7.05 (2.06)

Table 3 describes the proportion of DHT identified at screening and random selection stages. At screening 14 types of DHT were identified across all papers to be considered for full paper review (*n* = 821). VR (19.1%, *n* = 157), exoskeleton (17.9%, *n* = 147), and neuromuscular stimulation (12.7%, *n* = 104) were most prevalent and contributed to the half of the publications. The least commonly identified technologies were Vagus nerve stimulation (0.1%, *n* = 1), theta stimulation (0.1%, *n* = 1), vibration (0.1%, *n* = 1), balance platforms and apps (0.5%, *n* = 4).

**Table 3:** Type of DHT used across all papers screened and randomly selected for data analysis.

Technology category	All papers screened (*n* = 821)		Random selection (*n* = 87)	
	*N*	%	*N*	%
Applications	4	<1	0	0.0
Balance platform	7	<1	1	1.1
Biofeedback	16	1.9	2	2.3
Brain stimulation	91	11.1	13	14.9
Brain-computer interface	13	1.6	3	3.4
Electrical stimulation	33	4.0	12	13.8
Exoskeleton	147	17.9	11	12.6
Magnetic stimulation	48	5.8	5	5.7
Neuromuscular stimulation	104	12.7	10	11.5
Peripheral stimulation	9	1.1	0	0.0
Robotics	89	10.8	10	11.5
Telehealth	18	2.2	1	1.1
Theta stimulation	1	<1	0	0.0
Treadmill	36	4.4	2	2.3
Unclear ^a^	11	1.3	0	n/a
Vagus nerve stimulation	1	<1	1	1.1
Vibration	1	<1	0	0.0
Virtual Reality	157	19.1	15	17.2
Wearables	35	4.3	1	1.1

^a^ Articles with unclear technology at screening and abstract stage were reviewed and recoded if they were part of the random selection.

Among the papers included in the full-text review (*n* = 87), the highest proportions were observed among VR (17.2%, *n* = 15), brain stimulation (14.9%, *n* = 13), electrical stimulation (13.8%, *n* = 12), exoskeleton (12.6%, *n* = 11), neuromuscular stimulation (11.5%, *n* = 10), and robotics (11.5%, *n* = 89). Four types of DHT were not included in the random sample: apps, peripheral stimulation, theta stimulation and vibration.

### Descriptive results for reporting equity based on PROGRESS-plus criteria

**Figure 2:**
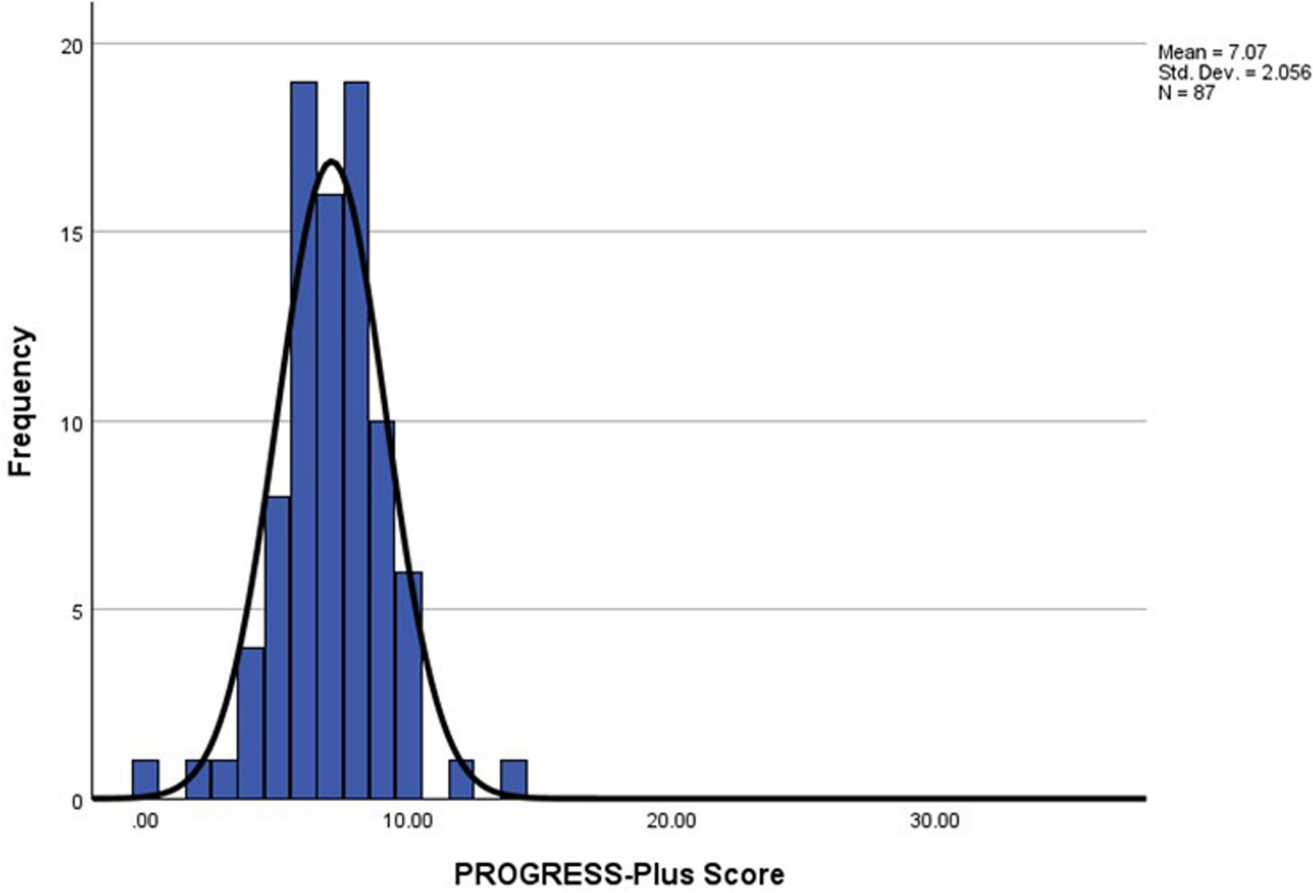
Distribution of total PROGRESS-Plus scores for included papers (*n* = 87).

The mean number of reported PROGRESS-Plus items used to describe participants was 7.05 (*SD* *=* 2.06, range: 0–14, see Figure 2) out of a possible 36 criteria.

**Figure 3:**
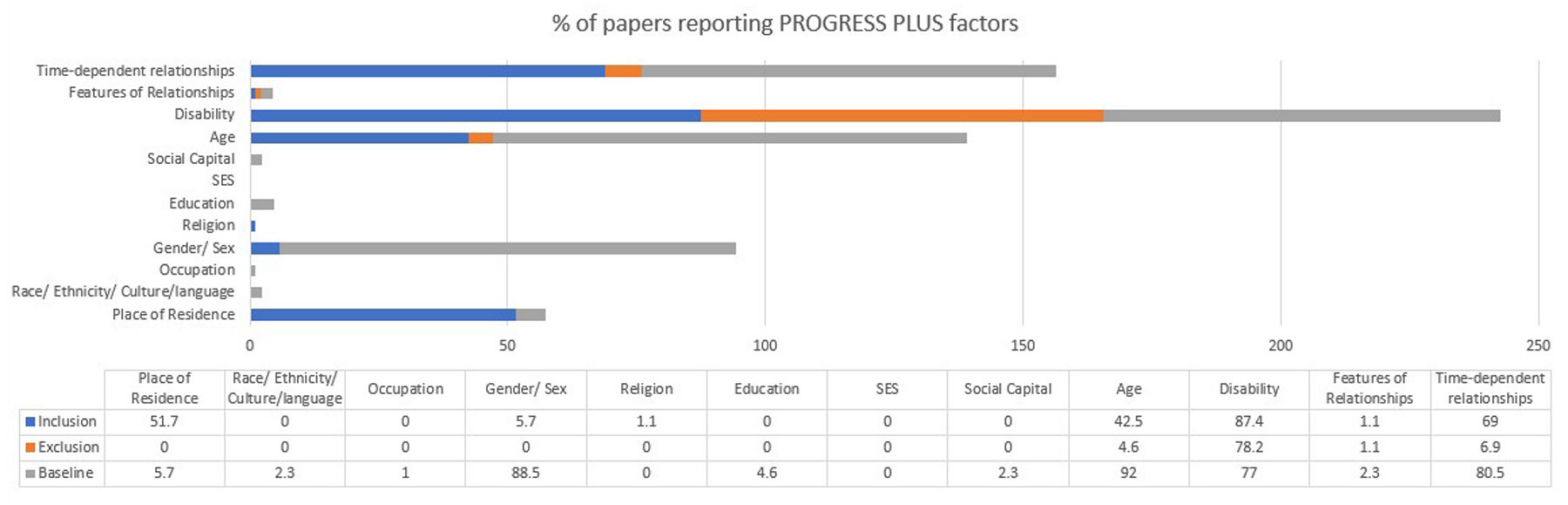
Proportion of papers reporting PROGRESS-plus criteria (*n* = 87).

`The most reported items were age as part of baseline characteristics (92%), disability as part of inclusion (87.4%) and exclusion criteria (78.2%) and at baseline (77%), gender at baseline (88.5%), and time-dependent relationships at inclusion criteria (69%) and at baseline (80.5%; see Figure 3). The time-dependent relationships focussed on time since stroke. Residence at inclusion was reported among 51.7% of the 87 papers e.g., at a specific hospital or region. Less than half of the papers included age as part of their inclusion criteria (42.5%) and only 4.6% included as part of their exclusion criteria. Most papers did not include any PROGRESS-Plus factors in exclusion criteria as shown in Figure 3.

Among the 36 equity factors, 15 factors (41.7%) were not reported in inclusion, exclusion or baseline characteristics (see Figure 3). Ethnicity, occupation, education, socioeconomic status and social capital were not reported in any inclusion criteria; place of residence, ethnicity, occupation, gender, religion, education, socioeconomic status and social capital were not reported in any exclusion criteria; and religion and socioeconomic status were not included in any baseline characteristics. Socioeconomic status was not reported in any inclusion, exclusion or baseline data.

### Variation for reporting equity factors based study characteristics

As shown in Table 4, there were no significant differences in PROGRESS-Plus scores by the location of the publication, *F* (4, 87) = .48, *p* = .75, the year of publication, *F* (4, 87) = .48, *p* = .75, the target of the intervention *F*(4, 87) = .48, *p* = .75, the number of comparison groups included in the trial *F*(4, 87) = .48, *p* = .75, the sample size and by the type of DHT tested *F*(4, 87) = .48, *p* = .75. No variation was explained through multiple linear regression factors associated with greater equity reporting. However, a positive small correlation (*r* = .26, *n* = 87, *p* < 0.05) was identified between the year of publication as a continuous variable and PROGRESS-Plus scores suggesting that the recency of publication was weakly and positively associated with an increase in PROGRESS-Plus score reporting.

**Table 4:** The results from ANOVA one-way analysis of variance in PROGRESS-plus scores.

Factor	PROGRESS-plus score	Degrees of freedom	F	*p*-value
	Mean (SD)			
Location		4	.48	.754
Europe	6.71 (1.59)			
South America	7.67 (1.53)			
North America	7.88 (3.09)			
Asia	7.00 (2.07)			
Africa	7.00 (1.73			
Year of publication		3	2.64	.078
2011–2015	6.45 (2.51)			
2016–2019	7.23 (1.78)			
2020–2021	7.81 (1.42)			
Intervention target		2	.838	.436
Lower extremity	7.12 (2.09)			
Upper extremity	6.88 (1.98)			
Both	8.25 (2.87)			
Number of comparison groups (including control)		2	.555	.576
Two groups	6.96 (2.15)			
Three groups	7.64 (1.57)			
Four groups	7.50 (0.71)			
Sample size		1	3.68	.058
30 or less participants	6.67 (1.87)			
31 or more participants	7.5 (2.18)			
Type of DHT ^a^		4	1.02	.404
(1) Tech with physical support	7.17 (1.71)			
(2) Forms of stimulation	7.68 (1.42)			
(3) Forms of electrical stimulation	6.64 (2.59)			
(4) Sensors and feedback	6.33 (3.27)			
(5) Tech with remote activity^b^	12.00			
(6) Virtual Reality	6.73 (1.33)			

^a^ DHT categories reduced from 14 to 6 categories as described in the data analysis section.

^b^ Excluded from the analysis due to having only 1 paper being included in this category.

## Discussion

Our review is the first, to our knowledge, to systematically describe the equity factors that are and are not, commonly reported in trials of DHT for stroke rehabilitation. Its findings indicate that most studies failed to consider even half of the PROGRESS-Plus factors, despite their importance to health outcomes.

There is longstanding recognition that medical care and rehabilitation after stroke face challenges both nationally and globally to ensure equity of access and outcome (28, 29). The use of DHT in rehabilitation seeks to provide efficacious care, with some technologies developed to overcome barriers that have traditionally limited access to rehabilitation services, e.g., geography, whilst others seek to improve outcomes. This systematic review sought to determine to what extent equity factors are being reported in stroke rehabilitation trials evaluating DHT and provide a foundation for further research to elucidate the reasons why some factors were reported whilst others were not.

Our findings from a random selection of 87 RCTs indicate that only 21 out of 36 equity factors derived from the PROGRESS-Plus were reported. Age, gender, disability, and time-dependent relationships (typically time since stroke) were the most common factors to be included in papers, although even these were not reported by all. Education, ethnicity, occupation, religion, social capital and socioeconomic status were reported by less than 5% of studies. With the exception of disability, which was commonly used as an exclusion criteria in the sampled studies, few equity factors were included in the inclusion or exclusion criteria, indicating that groups of participants were not being deliberately excluded from research participation. Coding of disabilities included criteria that excluded patients based on psychological capabilities (e.g., capacity to consent, mental illnesses etc.) or co-morbidities (e.g., cancer, implants etc.); it was likely that these criteria were utilised as they were perceived to potentially confound outcomes of the interventions under scrutiny. However, the lack of reporting of equity factors makes any other biases around participant selection difficult to detect**.**

Whilst few equity factors were included in inclusion or exclusion criteria, indicating that groups of participants were not being deliberatively excluded from research participation, the lack of reporting of equity factors makes any biases around participant selection difficult to detect. There was no single predictor that increased the likelihood of papers reporting equity factors, although there was a tendency for more recent papers to report more factors. This lack of inclusivity means that important findings relevant to different populations may be missed, study results may not be applicable in broader contexts, and we miss opportunities to understand the responses in different communities. Without clearly articulating equity factors, equity-relevant trials and tailored clinical interventions cannot be developed to benefit those who experience specific inequities (30). The absence of reporting and the resultant limitations to understanding in whom research findings can be confidently applied also affect clinical decision-making, reducing clinicians' confidence in the ability of research to improve practice. This not only significantly impacts the translation of findings to clinical practice, but also risks further increasing health inequity for many patients.

### Equity factors and DHT

There were no significant differences in the reporting of equity factors by the DHT tested, sample size of the studies, number of comparison groups, location, and the extremity being tested. Despite the absence of statistical significance, there was an observable trend for a greater number of equity factors being reported for telehealth interventions, whilst the least number of factors were reported for interventions using brain-computer interfaces (BCI) and Vagus nerve stimulation. This could be explained by the nature of the interventions—telehealth was explicitly designed to be based outside of acute healthcare settings, only requires commercial and readily available technologies (internet connectivity, computer, phone or tablet) and was relatively well established in the period of the review (a Cochrane review on its effectiveness was first published in 2013 and then repeated in 2020) (25); in contrast, relatively nascent technologies, such as BCI, require bespoke equipment, are more likely to be at a developmental stage and so tend occur in healthcare/laboratory settings rather in the community. The target of the intervention may also affect the perception of the importance of collecting equity data from participants; for example, rehabilitative BCI and vagal nerve stimulation seeks to stimulate neuronal circuits to improve motor impairments whilst telehealth typically offers multifaceted interventions considering aspects of impairment, activities and participation. As both activities and participation are heavily influenced by personal and environmental factors as well as impairments produced by stroke, it is possible that researchers sought to capture a wider range of social and personal factors, including some of those included on the PROGRESS-Plus tool, to identify potential confounding variables in these studies.

Although telehealth interventions tended to report more equity factors than other DHT studies, no studies reported all the factors that are associated with digital exclusion, namely age, disability, socioeconomic status and education. These factors also influence outcomes after stroke and so are important to capture in any rehabilitation studies, but particularly so in trials of DHT as age, disability, socioeconomic status and education also influence digital inclusion (6).

This review found that, despite its importance to digital inclusion and wider physical health, socioeconomic status was not reported in any studies (31). Education and occupation, which directly influence socioeconomic status, were reported by less than 5% of included papers. This is important as populations with lower socioeconomic status are more likely to have a stroke and so will form a significant part of the clinical population that rehabilitation research is supposed to impact. They are also likely to have poorer short and long-term outcomes after their stroke, and so exhibit greater need for healthcare and rehabilitation services, but will experience greater digital exclusion compared to those from higher socioeconomic backgrounds (29). Paradoxically, people who take part in research are typically educated, work in better paid jobs and are from higher socioeconomic groups (32), indicating that research does not occur in the groups where it is most needed (8) and widening the gulf between who participates in research and for whom research findings are intended. This potential mismatch between those who are likely to be involved in research and the wider patient population means that research findings have limited validity to clinical practice and cannot confidently be generalised to the majority of patients. Inconsistent and incomplete reporting of equity factors in DHT interventions may further exacerbate disparities in stroke care and patient outcomes. In England, low socioeconomic status has been associated with significantly greater risk of 1-year mortality and less likely to receive key care processes after stroke (33). The lack of reporting by patient's ethnicity, deprivation indicators such as education and occupation and other wider determinants of health in trials could hinder implementation of evidence in practice and ability to tailor/adopt interventions for specific patient populations. Furthermore, a recent statement from American Heart Association highlights the lack of studies in stroke rehabilitation that aims to address inequities in patient outcomes and the need to identify and quantify where inequities exist. This review further highlights an important gap in stroke rehabilitation research which may remain unaddressed without a systemic change in reporting and designing clinical trials (34).

One small encouraging finding from this review was that we identified a small positive association between the year of publication and the increase in PROGRESS-Plus scores reporting. This could indicate that recently published studies are more likely to consider recording and reporting more equity factors than that those published several years ago but would need to be replicated in future work.

### Limitations

The novel approach to this review has not been previously used in stroke rehabilitation research but builds on, and is informed by, other metascience or “research on research” in stroke which have used a sample of published evidence to represent a large research field (21). Using only a subset of papers means that we cannot be certain that the sample was representative of the larger field, although their random selection limited any systematic bias. Similarly, only including papers published between 2011 and 2021 could mean that changes in reporting of the equity factors before or after this period were missed. It is also worth noting that the exclusion of studies not written in English limits the generalisability of these findings to the wider evidence base and that, as disagreements between raters were resolved by discussions, the level of agreement between raters was not recorded.

Furthermore, as this review sought to describe the landscape for the equity factors that have been reported in DHT trials in stroke rehabilitation, it did not further explore why this might be the case using narrative synthesis in its analytical methods due to the heterogeneity of the DHT types, the variation in the intervention modalities and outcomes identified in this review. However, the reasons should be considered in future reviews with further focus on specific DHTs and the primary target of the intervention. We would suggest that qualitative and consensus-based research methodologies would be beneficial to understand how equity can be improved in line with the World Health Organisation's recommendations for improving effectiveness and equity in clinical trials (35).

Despite these limitations, the extracted data indicates a clear diversity in DHT, location and sample size providing confidence that the sample of papers in this limited period were largely representative of the larger field. Future studies can focus on specific technologies in stroke rehabilitation allowing improvements to be made in subject specific areas while this review describes a broader landscape in stroke rehabilitation trials using digital health technologies.

## Conclusions

Rehabilitation is based on the foundations of using evidence to train clinicians, guide practice, and inform service commissioning to produce optimal recovery for people after stroke. Our findings highlight that whilst pieces of key data that relate to both health equity and digital inclusion (age and disability) were captured in some stroke rehabilitation research evaluating DHT, other equally important data showing ethnicity and socio-economic status are not. The absence of reporting of equity data by most papers we reviewed highlights a blind spot in our knowledge of who is participating in research evaluating DHT in rehabilitation. This serious shortcoming means we cannot judge how representative the research participants, and by extension, the research findings, are of the wider clinical stroke rehabilitation population and precipitates a lack of comprehensive understanding of inequities in both research and practice. This lack of understanding hampers attempts to proactively reduce inequities and improve health outcomes. We argue that it is vital that both those who conduct and fund research ensure that equity data are collected routinely for all studies to recognise and address inequities. This is particularly pertinent for studies of DHT in stroke rehabilitation where health inequalities could be compounded by digital exclusion. Only through understanding and addressing inequities can we be confident that people after stroke have fair opportunities to be involved in research to improve their outcomes and ensure that research can be confidently used to guide clinical practice to benefit all.

## Data Availability

The original contributions presented in the study are included in the article/Supplementary Material, further inquiries can be directed to the corresponding author.
