# Supplementary material for: How equitable is digital rehabilitation for people after stroke? A systematic review using an equity approach
Source: Front Digit Health. 2025 Jun 24;7:1544754. doi: 10.3389/fdgth.2025.1544754 (PMC12234467; doi:10.3389/fdgth.2025.1544754)
Supplement: Supplementary file 2 [file Datasheet1.docx]

Equity Supplementary Materials

**Supplementary Materials 1 – Detailed search strategies**

Medline

| 1 | cerebrovascular disorders/ or exp basal ganglia cerebrovascular disease/ or brain ischemia/ or exp brain infarction/ or ischemic attack, transient/ or vertebrobasilar insufficiency/ or exp carotid artery diseases/ or cerebral small vessel diseases/ or cerebral amyloid angiopathy, familial/ or stroke, lacunar/ or cerebrovascular trauma/ or vertebral artery dissection/ or intracranial arterial diseases/ or cerebral arterial diseases/ or cerebral amyloid angiopathy/ or infarction, anterior cerebral artery/ or infarction, middle cerebral artery/ or infarction, posterior cerebral artery/ or moyamoya disease/ or intracranial aneurysm/ or intracranial arteriosclerosis/ or exp intracranial arteriovenous malformations/ or exp "intracranial embolism and thrombosis"/ or intracranial hemorrhages/ or exp cerebral hemorrhage/ or intracranial hemorrhage, hypertensive/ or exp subarachnoid hemorrhage/ or stroke/ or vasospasm, intracranial/ |
| --- | --- |
| 2 | stroke rehabilitation/ |
| 3 | Brain Injury, Chronic/ |
| 4 | (stroke* or cva* or poststroke or post-stroke or cerebrovasc* or (cerebr* adj3 vasc*)).tw. |
| 5 | ((cerebr* or cerebell* or vertebrobasil* or brain) adj3 (isch?emi* or infarct* or thrombo* or emboli* or apoplex*)).tw. |
| 6 | ((cerebr* or cerebell* or vertebrobasil* or subarachnoid* or arachnoid* or brain) adj3 (h?emorrhag* or h?ematom* or bleed*)).tw. |
| 7 | or/1-6 |
| 8 | Physical Therapy Modalities/ |
| 9 | Physical Therapy Specialty/ |
| 10 | rehabilitation/ or early ambulation/ or exp exercise therapy/ or exp neurological rehabilitation/ or telerehabilitation/ |
| 11 | Allied Health Occupations/ |
| 12 | Therapeutics/ |
| 13 | Physical Therapist Assistants/ |
| 14 | Allied Health Personnel/ |
| 15 | (Physiotherap* or physio therap* or physical therap* or physiatr* or neurorehabilitation or mobili#ation or motor activit* or motor skill* or ambulation or stimulation or mobility or training program* or Telerehab* or tele-rehab*).tw. |
| 16 | ((Functional or resistance or strength or weight or limb or balance or virtual reality or gait or stability) adj3 training).tw. |
| 17 | ((motor or sensorimotor or movement) adj3 recovery).tw. |
| 18 | or/8-17 |
| 19 | randomized controlled trial.pt. |
| 20 | controlled clinical trial.pt. |
| 21 | randomized.ab. |
| 22 | placebo.ab. |
| 23 | clinical trials as topic.sh. |
| 24 | randomly.ab. |
| 25 | trial.ti. |
| 26 | or/19-25 |
| 27 | exp animals/ not humans.sh. |
| 28 | 26 not 27 |
| 29 | 7 and 18 and 28 |
| 30 | limit 29 to (english language and yr="2011 -Current") |

Embase

| 1 | *cerebrovascular disease/ or exp *basal ganglion hemorrhage/ or exp *brain ischemia/ or exp *brain hemangioma/ or exp *brain hematoma/ or exp *brain infarction/ or *transient ischemic attack/ or *vertebrobasilar insufficiency/ or exp *carotid artery disease/ or *vascular amyloidosis/ or *lacunar stroke/ or *artery dissection/ or exp *cerebral artery disease/ or *moyamoya disease/ or exp *intracranial aneurysm/ or *brain atherosclerosis/ or exp *cerebrovascular malformation/ or exp *occlusive cerebrovascular disease/ or exp *thromboembolism/ or exp *brain hemorrhage/ or exp *subarachnoid hemorrhage/ or exp *cerebrovascular accident/ or *brain vasospasm/ |
| --- | --- |
| 2 | *stroke rehabilitation/ |
| 3 | (stroke* or cva* or poststroke or post-stroke or cerebrovasc* or (cerebr* adj3 vasc*)).tw. |
| 4 | ((cerebr* or cerebell* or vertebrobasil* or brain) adj3 (isch?emi* or infarct* or thrombo* or emboli* or apoplex*)).tw. |
| 5 | ((cerebr* or cerebell* or vertebrobasil* or subarachnoid* or arachnoid* or brain) adj3 (h?emorrhag* or h?ematom* or bleed*)).tw. |
| 6 | or/1-5 |
| 7 | *physiotherapy/ |
| 8 | *rehabilitation/ or *constraint induced therapy/ or *mirror therapy/ or *muscle training/ or *neurorehabilitation/ or *sensorimotor integration/ or *telerehabilitation/ |
| 9 | *physiotherapist assistant/ |
| 10 | (Physiotherap* or physio therap* or physical therap* or physiatr* or neurorehabilitation or mobili#ation or motor activit* or motor skill* or ambulation or stimulation or mobility or training program* or Telerehab* or tele-rehab*).tw. |
| 11 | ((Functional or resistance or strength or weight or limb or balance or virtual reality or gait or stability) adj3 training).tw. |
| 12 | ((motor or sensorimotor or movement) adj3 recovery).tw. |
| 13 | or/7-12 |
| 14 | Randomized controlled trial/ |
| 15 | Controlled clinical trial/ |
| 16 | random$.ti,ab. |
| 17 | randomization/ |
| 18 | intermethod comparison/ |
| 19 | placebo.ti,ab. |
| 20 | (compare or compared or comparison).ti. |
| 21 | ((evaluated or evaluate or evaluating or assessed or assess) and (compare or compared or comparing or comparison)).ab. |
| 22 | (open adj label).ti,ab. |
| 23 | ((double or single or doubly or singly) adj (blind or blinded or blindly)).ti,ab. |
| 24 | double blind procedure/ |
| 25 | parallel group$1.ti,ab. |
| 26 | (crossover or cross over).ti,ab. |
| 27 | ((assign$ or match or matched or allocation) adj5 (alternate or group$1 or intervention$1 or patient$1 or subject$1 or participant$1)).ti,ab. |
| 28 | (assigned or allocated).ti,ab. |
| 29 | (controlled adj7 (study or design or trial)).ti,ab. |
| 30 | (volunteer or volunteers).ti,ab. |
| 31 | human experiment/ |
| 32 | trial.ti. |
| 33 | or/14-32 |
| 34 | (random$ adj sampl$ adj7 (cross section$ or questionnaire$1 or survey$ or database$1)).ti,ab. not (comparative study/ or controlled study/ or randomi?ed controlled.ti,ab. or randomly assigned.ti,ab.) |
| 35 | Cross-sectional study/ not (randomized controlled trial/ or controlled clinical study/ or controlled study/ or randomi?ed controlled.ti,ab. or control group$1.ti,ab.) |
| 36 | (((case adj control$) and random$) not randomi?ed controlled).ti,ab. |
| 37 | (Systematic review not (trial or study)).ti. |
| 38 | (nonrandom$ not random$).ti,ab. |
| 39 | Random field$.ti,ab. |
| 40 | (random cluster adj3 sampl$).ti,ab. |
| 41 | (review.ab. and review.pt.) not trial.ti. |
| 42 | we searched.ab. and (review.ti. or review.pt.) |
| 43 | update review.ab. |
| 44 | (databases adj4 searched).ab. |
| 45 | (rat or rats or mouse or mice or swine or porcine or murine or sheep or lambs or pigs or piglets or rabbit or rabbits or cat or cats or dog or dogs or cattle or bovine or monkey or monkeys or trout or marmoset$1).ti. and animal experiment/ |
| 46 | Animal experiment/ not (human experiment/ or human/) |
| 47 | or/34-46 |
| 48 | 33 not 47 |
| 49 | 6 and 13 and 48 |
| 50 | limit 49 to (english language and yr="2011 -Current") |

CINAHL

| 1 | TI (animal model*) |
| --- | --- |
| 2 | MH (animal studies) |
| 3 | MH animals+ |
| 4 | AB (cluster W3 RCT) |
| 5 | MH (crossover design) OR MH (comparative studies) |
| 6 | AB (control W5 group) |
| 7 | PT (randomized controlled trial) |
| 8 | MH (placebos) |
| 9 | MH (sample size) AND AB (assigned OR allocated OR control) |
| 10 | TI (trial) |
| 11 | AB (random*) |
| 12 | TI (randomised OR randomized) |
| 13 | MH "cluster sample" |
| 14 | MH "pretest‐posttest design" |
| 15 | MH "random assignment" |
| 16 | MH "single‐blind studies" |
| 17 | MH "double‐blind studies" |
| 18 | MH "randomized controlled trials" |
| 19 | S7 OR S8 OR S9 OR S10 OR S11 OR S12 OR S13 OR S14 OR S15 OR S16 OR S17 OR S18 |
| 20 | TI ( (motor or sensorimotor or movement) N3 recovery ) OR AB ( (motor or sensorimotor or movement) N3 recovery ) |
| 21 | TI ( (Functional or resistance or strength or weight or limb or balance or "virtual reality" or gait or stability) N3 training ) OR AB ( (Functional or resistance or strength or weight or limb or balance or "virtual reality" or gait or stability) N3 training ) |
| 22 | TI ( Physiotherap* or "physio therap*" or "physical therap*" or physiatr* or neurorehabilitation or mobili?ation or "motor activit*" or "motor skill*" or ambulation or stimulation or mobility or "training program*" or Telerehab* or "tele-rehab*" ) OR AB ( Physiotherap* or "physio therap*" or "physical therap*" or physiatr* or neurorehabilitation or mobili?ation or "motor activit*" or "motor skill*" or ambulation or stimulation or mobility or "training program*" or Telerehab* or "tele-rehab*" ) |
| 23 | (MH "Allied Health Personnel") |
| 24 | (MH "Physical Therapist Assistants") |
| 25 | (MH "Therapeutics") |
| 26 | (MH "Allied Health Professions") |
| 27 | (MH "Telerehabilitation") |
| 28 | (MH "Therapeutic Exercise+") |
| 29 | (MH "Early Ambulation") |
| 30 | (MH "Rehabilitation") |
| 31 | (MH "Physical Therapy") |
| 32 | S1 OR S2 OR S3 OR S4 OR S5 |
| 33 | TI ( ((cerebr* or cerebell* or vertebrobasil* or subarachnoid* or arachnoid* or brain) N3 (h#emorrhag* or h#ematom* or bleed*)) ) OR AB ( ((cerebr* or cerebell* or vertebrobasil* or subarachnoid* or arachnoid* or brain) N3 (h#emorrhag* or h#ematom* or bleed*)) ) |
| 34 | TI ( ((cerebr* or cerebell* or vertebrobasil* or brain) N3 (isch#emi* or infarct* or thrombo* or emboli* or apoplex*)) ) OR AB ( ((cerebr* or cerebell* or vertebrobasil* or brain) N3 (isch#emi* or infarct* or thrombo* or emboli* or apoplex*)) ) |
| 35 | TI ( (stroke* or cva* or poststroke or "post-stroke" or cerebrovasc* or (cerebr* N3 vasc*)) ) OR AB ( (stroke* or cva* or poststroke or "post-stroke" or cerebrovasc* or (cerebr* N3 vasc*)) ) |
| 36 | (MH "Brain Damage, Chronic") |
| 37 | (MH "Cerebrovascular Disorders") OR (MH "Basal Ganglia Cerebrovascular Disease+") OR (MH "Carotid Artery Diseases") OR (MH "Carotid Artery Dissections") OR (MH "Carotid Artery Thrombosis") OR (MH "Carotid Stenosis") OR (MH "Cerebral Ischemia") OR (MH "Cerebral Ischemia, Transient") OR (MH "Cerebral Small Vessel Diseases") OR (MH "Cerebral Vasospasm") OR (MH "Cerebral Arterial Diseases+") OR (MH "Cerebral Aneurysm") OR (MH "Intracranial Arterial Diseases") OR (MH "Cerebral Arterial Diseases") OR (MH "Intracranial Arteriosclerosis") OR (MH "Intracranial Embolism and Thrombosis") OR (MH "Intracranial Thrombosis+") OR (MH "Intracranial Hemorrhage+") OR (MH "Stroke+") OR (MH "Vertebral Artery Dissections") |
| 38 | S6 AND S19 AND S42 |
| 39 | S6 AND S19 AND S42 |
| 40 | S41 NOT S40 |
| 41 | S20 OR S21 OR S22 OR S23 OR S24 OR S25 OR S26 OR S27 OR S28 OR S29 OR S30 OR S31 OR S32 OR S33 OR S34 |
| 42 | S38 NOT S39 |
| 42 | MH (human) |
| 44 | S35 OR S36 OR S37 |

Cochrane

| 1 | MeSH descriptor: [Cerebrovascular Disorders] this term only |
| --- | --- |
| 2 | MeSH descriptor: [Basal Ganglia Cerebrovascular Disease] explode all trees |
| 3 | MeSH descriptor: [Brain Ischemia] this term only |
| 4 | MeSH descriptor: [Brain Infarction] explode all trees |
| 5 | MeSH descriptor: [Ischemic Attack, Transient] this term only |
| 6 | MeSH descriptor: [Vertebrobasilar Insufficiency] this term only |
| 7 | MeSH descriptor: [Carotid Artery Diseases] explode all trees |
| 8 | MeSH descriptor: [Cerebral Small Vessel Diseases] this term only |
| 9 | MeSH descriptor: [Cerebral Amyloid Angiopathy, Familial] this term only |
| 10 | MeSH descriptor: [Stroke, Lacunar] this term only |
| 11 | MeSH descriptor: [Cerebrovascular Trauma] this term only |
| 12 | MeSH descriptor: [Vertebral Artery Dissection] this term only |
| 13 | MeSH descriptor: [Intracranial Arterial Diseases] this term only |
| 14 | MeSH descriptor: [Cerebral Arterial Diseases] this term only |
| 15 | MeSH descriptor: [Cerebral Amyloid Angiopathy] this term only |
| 16 | MeSH descriptor: [Infarction, Anterior Cerebral Artery] this term only |
| 17 | MeSH descriptor: [Infarction, Middle Cerebral Artery] this term only |
| 18 | MeSH descriptor: [Infarction, Posterior Cerebral Artery] this term only |
| 19 | MeSH descriptor: [Moyamoya Disease] this term only |
| 20 | MeSH descriptor: [Intracranial Aneurysm] this term only |
| 21 | MeSH descriptor: [Intracranial Arteriosclerosis] this term only |
| 22 | MeSH descriptor: [Intracranial Arteriovenous Malformations] explode all trees |
| 23 | MeSH descriptor: [Intracranial Embolism and Thrombosis] explode all trees |
| 24 | MeSH descriptor: [Intracranial Hemorrhages] this term only |
| 25 | MeSH descriptor: [Cerebral Hemorrhage] explode all trees |
| 26 | MeSH descriptor: [Intracranial Hemorrhage, Hypertensive] this term only |
| 27 | MeSH descriptor: [Subarachnoid Hemorrhage] explode all trees |
| 28 | MeSH descriptor: [Stroke] this term only |
| 29 | MeSH descriptor: [Vasospasm, Intracranial] this term only |
| 30 | MeSH descriptor: [Stroke Rehabilitation] this term only |
| 31 | MeSH descriptor: [Brain Injury, Chronic] this term only |
| 32 | (stroke* or cva* or poststroke or "post-stroke" or cerebrovasc* or (cerebr* NEAR/3 vasc*)):ti |
| 33 | (stroke* or cva* or poststroke or "post-stroke" or cerebrovasc* or (cerebr* NEAR/3 vasc*)):ab |
| 34 | ((cerebr* or cerebell* or vertebrobasil* or brain) NEAR/3 (isch?emi* or infarct* or thrombo* or emboli* or apoplex*)):ti |
| 35 | ((cerebr* or cerebell* or vertebrobasil* or brain) NEAR/3 (isch?emi* or infarct* or thrombo* or emboli* or apoplex*)):ab |
| 36 | ((cerebr* or cerebell* or vertebrobasil* or subarachnoid* or arachnoid* or brain) NEAR/3 (h?emorrhag* or h?ematom* or bleed*)):ti |
| 37 | ((cerebr* or cerebell* or vertebrobasil* or subarachnoid* or arachnoid* or brain) NEAR/3 (h?emorrhag* or h?ematom* or bleed*)):ab |
| 38 | {OR #1-#37} |
| 39 | MeSH descriptor: [Physical Therapy Modalities] this term only |
| 40 | MeSH descriptor: [Physical Therapy Specialty] this term only |
| 41 | MeSH descriptor: [Rehabilitation] this term only |
| 42 | MeSH descriptor: [Early Ambulation] this term only |
| 43 | MeSH descriptor: [Exercise Therapy] explode all trees |
| 44 | MeSH descriptor: [Neurological Rehabilitation] explode all trees |
| 45 | MeSH descriptor: [Telerehabilitation] this term only |
| 46 | MeSH descriptor: [Allied Health Occupations] this term only |
| 47 | MeSH descriptor: [Therapeutics] this term only |
| 48 | MeSH descriptor: [Physical Therapist Assistants] this term only |
| 49 | MeSH descriptor: [Allied Health Personnel] this term only |
| 50 | (Physiotherap* or "physio therapy" or "physio therapist" or "physical therapy" or "physical therapies" or "physical therapist" or physiatr* or neurorehabilitation or mobili#ation or "motor activity" or "motor activities" or "motor skill" or "motor skills" or ambulation or stimulation or mobility or (training NEXT program*) or Telerehab* or "tele-rehab" or "tele-rehabilitation"):ti |
| 51 | (Physiotherap* or "physio therapy" or "physio therapist" or "physical therapy" or "physical therapies" or "physical therapist" or physiatr* or neurorehabilitation or mobili#ation or "motor activity" or "motor activities" or "motor skill" or "motor skills" or ambulation or stimulation or mobility or (training NEXT program*) or Telerehab* or "tele-rehab" or "tele-rehabilitation"):ab |
| 52 | ((Functional or resistance or strength or weight or limb or balance or "virtual reality" or gait or stability) NEAR/3 training):ti |
| 53 | ((Functional or resistance or strength or weight or limb or balance or "virtual reality" or gait or stability) NEAR/3 training):ab |
| 54 | ((motor or sensorimotor or movement) NEAR/3 recovery):ti |
| 55 | ((motor or sensorimotor or movement) NEAR/3 recovery):ab |
| 56 | {OR #39-#55} |
| 57 | #38 AND #56 |
| 58 | #38 AND #56 with Publication Year from 2011 to present, in Trials |

**Supplementary Materials 2 – PROGRESS-Plus definitions used for coding**

| **PROGRESS-Plus** | **Definition** |
| --- | --- |
| **Place of residence: Count** (no = 0, yes = 1) | Residence in a medically underserved area where it is difficult to access care. Example: rural, remote, inner cit |
| **Inclusion criteria** | This could be if the location where they have been recruited to the trial. For instance. Patients were recruited from the X hospital in Y city. |
| **Exclusion criteria** |  |
| **Baseline characteristics** |  |
| **Race/ethnicity/culture/language: Count** (no = 0, yes = 1) | Ethnic and racial minorities, patients who do not speak the dominant language of the region or who do not identify with the dominant culture of the area. |
| **Inclusion criteria** |  |
| **Exclusion criteria** |  |
| **Baseline characteristics** |  |
| **Occupation: Count** (no = 0, yes = 1) | Occupations that involve high risk exposures or unsafe working environments, instability in employment status, lack of access to employee benefits or employerfunded insurance systems. Example: part-time, disability leave, temporary worker, migrant worker |
| **Inclusion criteria** |  |
| **Exclusion criteria** |  |
| **Baseline characteristics** |  |
| **Gender/sex: Count** (no = 0, yes = 1) | Gender roles that may define differential access to health services and differential exposure to health risks, sexual identities that face violence and discrimination. Example: men, women, cisgender, transgender, intersex |
| Inclusion criteria |  |
| Exclusion criteria |  |
| Baseline characteristics |  |
| **Religion: Count** (no = 0, yes = 1) | Religious beliefs may limit a patient’s ability to choose certain medical therapies, religious affiliations may lead to discrimination and bias from service providers. |
| Inclusion criteria |  |
| Exclusion criteria |  |
| Baseline characteristics |  |
| **Education: Count** (no = 0, yes = 1) | Education level and education opportunities correlate with income status as well as knowledge about health and access to preventative health practices. Example: highest level of education completed, education status of family members |
| Inclusion criteria |  |
| Exclusion criteria |  |
| Baseline characteristics |  |
| **Socioeconomic status: Count** (no = 0, yes = 1) | Income levels that allow or prohibit participation in preventative health behaviours, ability to access health insurance in times of illness. Example: low income, private health insurance, state-sponsored insurance |
| Inclusion criteria |  |
| Exclusion criteria |  |
| Baseline characteristics |  |
| **Social capital: Count** (no = 0, yes = 1) | Social relationships and availability of social support networks to provide support and build resilience in times of distress. Example: marital status, community networks, professional networks |
| Inclusion criteria |  |
| Exclusion criteria |  |
| Baseline characteristics |  |
| **Plus Age: Count** (no = 0, yes = 1) | Old age and frailty may be associated with decreased independence, decreased social capital, and increased health comorbidities; young age may be associated with decreased decision-making power. Example: elderly or young |
| Inclusion criteria |  |
| Exclusion criteria |  |
| Baseline characteristics |  |
| **Plus Disability: Count** (no = 0, yes = 1) | Any mental health assessment, any quality of life or functional assessment, as well as any comorbid condition that is explicitly severe enough for us to reasonably believe that it impacts the ability to self-manage. Example: mental health issues, intellectual disabilities, chronic pain, blindness, endstage renal disease, symptomatic heart dise |
| Inclusion criteria |  |
| Exclusion criteria |  |
| Baseline characteristics |  |
| **Plus Features of relationships: Count** (no = 0, yes = 1) | Relationships that impact an individual’s ability to assert their autonomy and selfmanage. Example: social hierarchies at school, work, or home |
| Inclusion criteria |  |
| Exclusion criteria |  |
| Baseline characteristics |  |
| **Plus Time dependent relationships: Count** (no = 0, yes = 1) | Times of transition where an individual may face increased risks for poor health management. Example: discharge from hospital, release from prison, students on the move, practice guideline changes |
| Inclusion criteria | This could be whether or not they specify time since stroke as part of their inclusion and exclusion |
| Exclusion criteria |  |
| Baseline characteristics | Likewise this is often time since stroke experienced |
